# Teaching the Principles of Pediatric Critical Care to Non-Intensivists in Resource Limited Settings: Challenges and Opportunities

**DOI:** 10.3389/fped.2018.00044

**Published:** 2018-03-02

**Authors:** Michael F. Canarie, Asha N. Shenoi

**Affiliations:** ^1^Department of Pediatrics, Yale University School of Medicine, New Haven, CT, United States; ^2^Department of Pediatrics, University of Kentucky, Lexington, KY, United States

**Keywords:** critical care, resource limited, training programs, pediatric, challenges

## Introduction

It is a dismal reality of global health that the vast majority of critically ill or injured children are found in regions of the world least equipped to care for them. Most of these severely ill or injured children are cared for in clinics, hospital wards, or, when available, adult intensive care units (ICUs) by providers with variable amounts of training. This lack of training may, in fact, play a significant role in the premature demise of children <5 years old, since millions of these deaths are felt to be preventable with the resources available (1). Evidence shows that even in a setting with constrained resources, early recognition, and prompt, decisive intervention may reduce mortality (2–4). It seems intuitive, therefore, that training of non-intensivists that focuses on these principles might improve the outcomes in critically ill children. How can this instruction be best achieved in areas where it is most needed? In this article, we review the benefits and challenges of implementing short-term curricula to teach the basic principles and practice of critical care medicine in resource limited settings (RLS).

## Current State of the Care of the Critically Ill Child

The essential role of community and preventative healthcare in promoting the well-being of children in RLS is well established and assumed here. Yet, improvements in primary and preventive care do not eliminate need for hospital-based care. A high percentage of children (an estimated 12–34%) seen in ambulatory settings, for example, are felt to require hospital assessment and/or admission (5). Multiple studies have shown mortality inversely related to distance from a hospital and that prehospital/emergent or advanced care resources are most limited where the majority of children die (6–8). In addition, we know that a growing percentage of preventable deaths result from the “neglected burden” of trauma; approximately 90% global trauma deaths occur in low- and middle-income countries (LMICs) and road deaths alone kill more than 200,000 children per year in RLS (9–11). All these ill or injured children require some (and perhaps an increasing) degree of medical care and demise is, in many cases, related to an inability to deliver timely or appropriate care (12).

Yet for those patients who require and receive hospital-level care, mortality remains high, with infection and sepsis often the common final pathway (13). A study from Guinea–Bissau observed that 25% of childhood deaths occurred after admission to a regional hospital (14). In general, the sparse available data cite a pediatric inpatient mortality rate between 12 and 50% in RLS, with a high percentage of those occurring during the first 24 h following admission (9, 15). This latter observation underscores the belief that treatment is often delayed in these settings for children arriving at care facilities after the onset of critical illness. It is not a foregone conclusion, however, that mortality for these hospitalized patients has improved over time. For instance, a large, retrospective study on pediatric sepsis admissions to Brazilian hospitals from 1992 to 2006 showed an overall reduction in cases of sepsis, but no improvement in the mortality rate of 19% (16, 17).

What is clear is that most critically ill children who survive to inpatient care facilities are treated in clinics, hospital wards, and infrequently in mixed ICUs by caregivers with variable amount of pediatric or advanced care training. In Nigeria, for example, there are only 380 ICU trained nurses in a country of 140 million people (17). In reality, critical care is just the continuum of care provided to any child with a life-threatening illness or injury beginning with the time of presentation to a health care facility (18). Where and by whom care is provided may go a long way in determining outcome.

## The Role of Critical Care Training

There is evidence that the presence of pediatric intensive care units (PICUs) and trained critical care physicians (intensivists) improves patient care and saves lives in both high resource and RLS (19–22). It is likely that the mortality benefit from intensive care is attributable to an integration of multiple elements including basic infrastructure, essential supplies, and equipment, training, and staffing. In practical terms, however, “intensive care” can only be provided where these substantial resources and trained, multidisciplinary personnel are in place. The mere presence of an “intensive care unit” does not guarantee the presence of intensive, integrated care, or good outcomes, as the mortality rates in mixed and exclusively pediatric ICUs in LMICs can be as high as 50–58% (22, 23). In any case, ICUs are uncommon in many parts of the world and, with rare exception, most of the world’s sickest children are cared for outside of conventional ICUs (pediatric or mixed) by caregivers without pediatric or critical care training.

How can the critical care of these children be improved? To begin, in RLS, critical care capacity is “developed” not “created” (24–26). This is not just a matter of semantics, but underlines the importance of a gradual cultivation and blending of training and technical capacity over time, rather than the creation of a physical space. Ultimately, there are two intertwining aspects of this undertaking: training and educational enrichment, and developing physical/technological ICU capacity to facilitate care. This is a delicate balance because most would agree that the introduction of material assets without concomitant thoughtful, systemic training is a futile endeavor. In fact, the lack of trained personnel in sufficient numbers is routinely cited as a weakness of healthcare delivery—often even more than inadequate supplies and equipment (26–28). Accordingly, training programs/courses that include a range of health care providers and focus on principles of early recognition and management of severe illness could play an important role in combatting healthcare-associated mortality.

## Standardized Critical Care Course: Why? What, and Where are They?

Standardized emergency and pediatric critical care curricula such as emergency, triage, assessment and treatment (ETAT), pediatric basic assessment and support intensive care (BASIC), and pediatric fundamentals of critical care study (PFCCS) are among of the educational initiatives used for supporting training in RLS (29). These courses provide a number of advantages even if they require some degree of “recalibration” to be effective in RLS (30). Standardized teaching modules are readily deployable, consistent, and comprehensive. They can also be shared and implemented for various training levels in a range of settings, reducing needless, and wasteful redundancy (31, 32). Tools such as the pediatric, emergency, assessment, recognition and stabilization (PEARS), and pediatric advanced life support (PALS) have even been bundled and used on system-wide levels to improve the care of sick children in Botswana and India (33).

Pediatric BASIC and PFCCS, both modeled on adult courses, have been specifically designed to teach non-intensivists the essential principles of care for the critically ill (32). The aim of these courses is to serve as a resource for those interested in learning to recognize critical illness and initiate care in the absence of an intensivist. As such, the courses focus on early identification of the critically ill child, initial steps in timely resuscitation, and organ support, and includes the rudiments of mechanical ventilation. Typically 2–3 days in duration, the sessions mix formal didactics, lectures, simulations, and practical sessions. Pretests are administered and the formal assessments at the end of the courses determine whether certificates are awarded to participants.

Pediatric fundamentals of critical care study is licensed by Society of Critical Care Medicine (SCCM). It can be administered either through a traditional live, instructor-led course, directed by a certified PFCCS director, or online. There is a well-defined process and oversight for certification of instructors and directors and periodic revision of content by an appointed task force. Although the course material covered can vary (based on the setting or participants’ needs), the contents of particular lectures are not modifiable. There has been a significant increase in international PFCCS courses recently, and between 2013 and 2016, more than 30 courses per year have been conducted around the world. There are currently 32 counties that have international PFCCS sites. SCCM has made significant effort to make the course affordable by having an option for tiered pricing based on a country’s gross domestic product at Purchasing Power Parity Per Capita but still the minimum cost is $630. However, in the absence of consistent external funding, even this subsidized rate can make access in RLS prohibitive.

Pediatric BASIC is another standardized critical care course created by pediatric critical care educational leaders during the 2011 World Congress of Pediatric Intensive and Critical Care Societies (WFPICCS). Its creators sought to design a course to teach the fundamentals of critical care in a format that was flexible and affordable and thus suitable for both high-income countries and RLS. This course, endorsed by WFPICCS, is run by volunteer pediatric critical care faculty from around the world in select places with strong local support. Target audience include mainly non-intensivist physicians and pediatric trainees (many with limited PICU experience), emergency medicine physicians, and senior PICU nurses. The development of Pediatric BASIC is overseen by a steering committee with expertise in pediatric critical care. No member of the steering committee, the original writers of the course, the instructors, or their families receive any financial benefit from the use of the course material and no individual owns intellectual property. Since there are no proprietorial fees associated with the course, it is always offered free of charge.

In addition, to meet local needs, the BASIC course allows flexibility to adapt not only the shape and content of the overall course but also individual lectures. Within the course, there is also an effort to do needs assessment at the training facility, soliciting input, and feedback from local faculty. While BASIC also has separate foundation courses for training nurses, in some institutions, nurses can also cotrained in the standard course. Since its inception in 2011, this course has been conducted in 63 different sites across 17 countries on 5 continents and a total of 1,617 providers have been trained. Pediatric BASIC has also shown success in building sustainable local critical care capacity through a “train the trainer” model in select institutions in countries such as India, Trinidad and Tobago, and Barbados. The first course in India was conducted in January 2014 and over next 4 years several local trainers (mostly Pediatricians with ICU experience) were trained as trainers using the model.

## Standardized Course: Evidence and Challenges

There is little research on the utility of short-term training programs, a deficiency not unique to RLS. Such programs, even in high-income countries, have a thin evidentiary underpinning, trying to answer three fundamental questions: Are the courses an effective way to transfer knowledge? Is this knowledge retained for any meaningful period? Finally, does this retained knowledge, in the end, translate into clinical benefit (34). Often data on “softer” or surrogate measures or outcomes are all that are available. Data on a decade of PALS courses in Israel, for example, revealed only participant satisfaction with the course and satisfactory completion by a high percentage of participants (35). Residents in Canada performed well on the PALS posttest, but not on the technical skills assessment, and showed poor retention at 12 months (36).

The lack of data bases in RLS makes it difficult to assess training programs of any kind. However, several studies have looked at various parameters in short-term courses. For instance, there is evidence that short training courses improve short-term knowledge in emergency and critical care (37, 38). Short courses teaching trauma care have been shown to identify deficiencies, increase provider skills, and improve trauma outcomes—including mortality—in developing countries like Trinidad, India, Ecuador, and Tanzania (30, 39–42). In fields such as pediatric surgery and obstetrics, short-term, specialized training courses have shown some success in knowledge retention (43, 44).

Standardized adult critical care programs have also been evaluated, both as educational instruments and as a means of transmitting knowledge (32). Training of Kenyan physicians using the Fundamental Critical Care Study (FCCS) course has been shown to increase the knowledge and confidence of new critical care skills (45). FCCS course participants in Zambia and Kenya felt that the material was site-appropriate and demonstrated an increase in clinical knowledge and confidence with procedures (46). There is less experience and evidence for pediatric standardized critical care curricula in RLS. However, a recent evaluation of Pediatric BASIC program in Northern Haiti showed post-course improvement in participants’ ability to manage patients in all topics covered by the course. The topics which showed the greatest improvement were related to support for respiratory failure using noninvasive and invasive ventilator support as well as the interpretation of blood gases (Silverman AMP, Napolitano et al., unpublished data, 2017).

The issue of course evaluation begs the very important question of the appropriateness of these tools in RLS. Given the range of disease and variable shortages of materiel encountered in RLS, training programs developed and taught by physicians from higher resourced countries may lack relevance (47). Consider, for instance, the teaching of PALS in places without defibrillators, or only a fraction of the recommended medications. It is, therefore, essential that courses should be tailored to local needs and, to assure ongoing relevance, have feedback mechanisms allowing for new data to inform future course modifications (30, 48).

Cost-effectiveness analysis is another important consideration, since optimizing the effects of expenditures takes on greater significance in regions where such funds are scarce. This type of evaluation, however, is complicated by a general dearth of data as well as uncertainty over relevant outcomes. Although the BASIC course itself is free, accurately measuring the actual cost of the “inputs” (i.e., concrete and opportunity costs of volunteer staff, cost to the facilities, etc.) would be difficult. In fact, the significance of the burden borne by the host institution could be hard to contextualize. In any case, without funding for educational programs, organizing courses may be overly burdensome to partners in RLS, threatening the feasibility and sustainability of these initiatives.

Another challenge to teaching these courses is the task of simulating the core critical care concepts of teamwork and collaborative learning in settings where these are not commonly practiced (49). In some cases, these difficulties may be due to cultural and hierarchical attitudes, but, nevertheless, could hinder training in optimal resuscitation and ongoing care. Nurses and ancillary personnel have variable levels of education and training and little experience with programs that train and integrate personnel at different levels concurrently (50).

Language can present a further practical barrier, limiting the effectiveness of these courses (51). Recent data from Haiti suggest that even with variable levels of skill in English and the use of translators, language problems were seen to limit course effectiveness (Silverman AMP, Napolitano et al., unpublished data, 2017). PFCCS course materials have been translated into Japanese, Spanish, and Chinese. BASIC is currently only available in English, but plans are underway to translate the teaching material into French and Spanish, allowing greater applicability.

In the end, assumptions on the benefits of training may have to suffice pending further evidence. Achieving a sustainable teaching model, however, through a “train the trainer” approach could assure long-term educational gains (48). Over time, steadfast application and consistent reinforcement may show the greatest benefits (33, 52). Yet this strategy will only be possible in settings with strong local interest and support.

## Conclusion

Training in the recognition and initial management of critical illness and injury may be an effective way to save the lives of countless children in RLS. Standardized courses in the fundamentals of critical care could be an effective component of this overall effort, helping to fill in gaps of trained personnel. Such courses should be adapted to local needs and resources. Also, while maintaining a consistent and readily reproducible, comprehensive curriculum, these courses should incorporate the feedback necessary to keep them locally relevant. In spite of the many challenges, standardized courses provide an opportunity to train large numbers of diverse personnel and achieve sustainability by instructing local trainers. Identifying relevant outcomes and gathering data will be an essential aspect of assuring future effectiveness and relevance.

## Author Contributions

AS and MC wrote the draft together and revised the contents. MC is primarily responsible for parts 1–3 and AS wrote the rest of the manuscript. Both authors revised the manuscript several times based on the feedback received from the coauthor.

## Conflict of Interest Statement

The authors declare that the research was conducted in the absence of any commercial or financial relationships that could be construed as a potential conflict of interest. The reviewer SC and handling Editor declared their shared affiliation.
